# Pharmacological Mechanisms of Phytochemicals and Pharmaceutical Agents in Protecting Against Methotrexate‐Induced Liver Injury

**DOI:** 10.1155/omcl/9407932

**Published:** 2026-04-09

**Authors:** Bushra Zia, Mouza Hasan Alqaishi Alshehhi, Azimullah Sheikh, Samir Mirza, Sandeep B. Subramanya, Shreesh K. Ojha

**Affiliations:** ^1^ Department of Pharmacology and Therapeutics, College of Medicine and Health Sciences, United Arab Emirates University, Al Ain, UAE, uaeu.ac.ae; ^2^ Department of Chemistry, College of Science, United Arab Emirates University, Al Ain, UAE, uaeu.ac.ae; ^3^ Department of Physiology, College of Medicine and Health Sciences, United Arab Emirates University, Al Ain, UAE, uaeu.ac.ae; ^4^ Zayed Centre for Health Sciences, United Arab Emirates University, Al Ain, UAE, uaeu.ac.ae

**Keywords:** chemotherapy-induced-toxicity, liver toxicity, methotrexate, natural products, protective agents

## Abstract

Methotrexate (MTX) is a widely used chemotherapeutic and immunosuppressive agent for treating various malignancies, autoimmune diseases (AIDs), and inflammatory disorders. Despite its therapeutic efficacy, long term or high‐dose MTX treatment is associated with a significant risk of hepatotoxicity, leading to MTX‐induced liver injury (MTX‐LI). The pathogenesis of MTX‐LI involves multiple mechanisms, including oxidative stress, mitochondrial dysfunction, inflammation, and altered metabolic processes, which collectively contribute to hepatocellular damage and fibrosis. Clinically, MTX‐LI manifests as elevated liver enzymes, hepatic steatosis, and, in severe cases, cirrhosis, posing a challenge to treatment regimens. To mitigate MTX‐LI, a growing body of research has focused on exploring therapeutic and/or preventive potential and pharmacological mechanisms of phytochemicals and pharmaceuticals. Phytochemicals, including flavonoids, terpenoids, alkaloids, and polyphenols, exhibit hepatoprotective effects attributed to their antioxidant, anti‐inflammatory, and antiapoptotic properties that can counteract MTX‐induced hepatic damage. Additionally, various pharmaceutical agents possessing antioxidants and anti‐inflammatory properties and favorably modulate metabolic pathways have shown promise in reducing the severity or progression of of MTX‐LI. The phytochemicals or pharmaceuticals showed beneficial in MTX‐LI primarily act by scavenging reactive oxygen species (ROS), modulating inflammatory pathways, and improving liver regeneration. Integrated together, its apparent that naturally occurring many phytochemicals as well as synthetic agents of pharmaceutical relevance are capable of preventing or alleviating MTX‐LI. However, the optimal strategies for integrating these agents into clinical practice require further investigations to highlight safety and efficacy in humans followed by pharmacological rationale of their possible use in therapeutics. Future directions should focus on elucidating the precise molecular mechanisms, establish safety and efficacy in humans, conducting regulatory toxicology studies and randomized clinical trials, and developing combination therapies for promotion as protective agents or adjuvants to maximize efficacy and minimize adverse effects.

## 1. Introduction

### 1.1. Overview of Methotrexate (MTX)

Methotrexate (MTX), pharmacologically a folate antagonist, is well used for a wide spectrum of clinical indications, including leukemia, lymphoma, breast cancer, rheumatoid arthritis (RA), and psoriasis, owing to its immunosuppressive and antineoplastic properties. MTX effectively inhibits cell proliferation by interfering with the production of purines and pyrimidines, making it an effective chemotherapeutic agent for treating cancer [1]. MTX is a cornerstone in the treatment of hematologic malignancies, such as acute lymphoblastic leukemia (ALL) and non‐Hodgkin lymphoma as well as solid tumors like osteosarcoma, breast cancer, and head and neck cancers [2–4]. The ability of MTX to affect both rapidly proliferating cancer cells and immune cells is what underscores its use in a variety of diseases. Moreover, MTX is also used to treat ectopic pregnancies, where the fertilized egg implants outside the uterus since MTX inhibits trophoblastic cell division, halting the growth of the embryo and preventing further complications [5]. Furthermore, MTX also finds its off‐label uses in treating numerous autoimmune diseases (AIDs) including scleroderma, sarcoidosis, and multiple sclerosis.

MTX primarily exerts its therapeutic effects by inhibiting the enzyme dihydrofolate reductase (DHFR), which is essential for converting dihydrofolate to tetrahydrofolate, a cofactor necessary for the synthesis of nucleotides, vital in the synthesis of DNA and RNA [6]. However, MTX does not solely inhibit DHFR but also may actively modulate its expression. Under normal physiological conditions, DHFR binds either to its co‐factor, nicotinamide adenine dinucleotide phosphate (NADPH), or to its corresponding mRNA, which limits DHFR synthesis. Since these two forms exist in equilibrium, competitive binding of MTX to DHFR may cause the dissociation of DHFR from its mRNA, thereby upregulate DHFR expression and potentially contribute to drug resistance [7]. The role of MTX in high‐dose chemotherapy regimens, either alone or in combination with other drugs has also been well‐established. MTX also regulates the activity of lymphocytes, contributing to its role in treating AIDs by suppressing abnormal immune responses. MTX inhibits the enzyme 5‐aminoimidazole‐4‐carboxamide ribonucleotide transformylase (AICAR transformylase), which plays an important role in purine biosynthesis. This inhibition leads to the accumulation of AICAR, which subsequently inhibits AMP deaminase. Consequently, a rise in the levels of adenosine contributes to the anti‐inflammatory actions of MTX through the immunosuppressive properties of adenosine [8]. In certain AIDs such as RA, psoriasis, and inflammatory bowel disease (IBD), MTX exerts its effects by inhibiting the proliferation of autoimmune T‐cells as well as reducing the binding of interleukin‐1β to its receptor, thereby resolving inflammation [9]. These actions make MTX a disease‐modifying antirheumatic drug (DMARD) in managing chronic autoimmune conditions and considered a preferred agent for the treatment of RA [10].

Despite the effectiveness of MTX in treating various conditions, its use is not devoid of significant adverse effects, particularly with long‐term therapy. The most common side effects of MTX include gastrointestinal issues like nausea, vomiting, and mucositis, as well as haematological effects such as bone marrow suppression, leading to pancytopenia (a reduction in red and white blood cells and platelets) [11, 12]. Teratogenicity, pulmonary toxicity, and renal impairment are some of the additional adverse effects associated with the administration of MTX during pregnancy [13, 14]. Among, numerous adverse drug reactions, hepatotoxicity is one of the most significant concern with the use of MTX, provided the crucial role of liver in metabolizing MTX, hence its accumulation may lead to the onset of pathogenic events including oxidative stress, inflammation, and apoptosis, all of which culminate into the hepatocellular damage and cell death [11].

In the early stages, mild liver injury may be seen as elevations in liver enzymes (AST, ALT), but these can progress to more serious conditions, such as cirrhosis, if MTX use is prolonged or at high doses [15]. Additionally, MTX‐associated liver fibrosis may develop over time, especially in individuals with risk factors like alcohol abuse, obesity, T2DM, and chronic liver disease. Hence, it is imperative to understand that MTX alone may not be the sole etiological factor in hepatotoxicity; rather, it may arise from a complex interplay of factors, including lifestyle influences and genetic predispositions [16].

Regular monitoring of liver function by measuring serum enzyme levels is crucial to detect early signs of liver injury in patients receiving MTX therapy. To avoid irreversible liver damage, it may occasionally be necessary to lower the MTX dosage or switch to alternative therapies. Hepatotoxicity remains a major concern with MTX therapy, as the liver continues to be the organ most frequently affected, posing a significant challenge to its long‐term use.

### 1.2. Pathophysiology of Liver Injury (MTX‐LI)

Liver injury (LI) can also be induced by numerous factors, encompassing both exogenous (external) and endogenous (internal) agents, with alcohol consumption being one of the most common causes of liver damage, leading to conditions ranging from non‐alcoholic fatty liver disease (NAFLD) to cirrhosis. Chronic alcohol intake triggers oxidative stress and the accumulation of acetaldehyde, a toxic metabolite that damages hepatocytes and promotes inflammatory responses [17]. NAFLD, which is associated with obesity, insulin resistance, and type 2 diabetes mellitus (T2DM) is another significant risk factor for liver injury.

In NAFLD, excess visceral fat leads to the accumulation of free fatty acids in hepatocytes, triggering inflammation, hepatocellular injury, and fibrosis thereby exacerbating the progression from simple fatty liver to non‐alcoholic steatohepatitis (NASH) and, ultimately, cirrhosis which is a critical aspect of NAFLD‐related liver disease [18]. Individuals with AIDs, such as autoimmune hepatitis, primary biliary cirrhosis, and primary sclerosing cholangitis, are at increased risk for liver injury due to abnormal immune responses that target liver cells and bile ducts, resulting in inflammation, liver damage, and fibrosis [19]. Medications such as antibiotics, acetaminophen, and statins, can also be hepatotoxic in some individuals, triggering drug‐induced liver injury (DILI), which can range from mild, transient liver enzyme elevation to acute liver failure [20]. Furthermore, viral infections, such as hepatitis B and hepatitis C, contribute significantly to liver injury by inducing chronic inflammation and fibrosis, eventually leading to cirrhosis and liver cancer [21].

MTX‐induced liver injury (MTX‐LI), a subset of DILI, is a complex and multifactorial condition resulting from both direct and indirect effects of the drug on the normal physiological state of hepatocytes. As has been discussed, liver is the main organ responsible for the metabolism and clearance of many drugs, including folate antagonist; MTX. The pathophysiology of MTX‐induced hepatotoxicity involves several interconnected mechanisms that may contribute to hepatocyte injury. One key mechanism is the production of free reactive radicals that may culminate into a state of oxidative stress since MTX is metabolized by the liver to its polyglutamated forms, which further interferes with cellular metabolism and increases the production of reactive oxygen species (ROS) [22]. These ROS damage lipid membranes, proteins, and DNA, initiating a series of cascade reactions that cause oxidative damage which further impairs cellular function. Mitochondria are particularly susceptible to oxidative stress, and their dysfunction, which leads to ATP depletion, is a hallmark of MTX‐LI [22]. This mitochondrial dysfunction can cause the release of cytochrome c, leading to the apoptosis of hepatocytes and their cellular degeneration.

Another significant contributor to MTX‐LI is inflammation, since MTX induces the release of pro‐inflammatory cytokines, including tumor necrosis factor‐alpha (TNF‐α), interleukin‐6 (IL‐6), and interleukin‐1β (IL‐1β), which activate the nuclear factor‐kappa B (NF‐κB) pathway and promote further inflammation [23]. This cascade of inflammatory events enhances liver cell damage and fibrosis. Furthermore, chronic MTX exposure has been shown to activate hepatic stellate cells (HSCs), leading to fibrogenesis and ultimately liver fibrosis in susceptible individuals. These mechanisms underscore the multifactorial nature of MTX‐induced hepatotoxicity, which can progress from mild elevations in liver enzymes to more severe outcomes such as cirrhosis and even liver failure in extreme cases.

The clinical manifestations of MTX‐LI can vary widely, depending on the severity of the damage. In its early stages, MTX‐LI may present with mild, asymptomatic elevations in liver enzymes (alanine aminotransferase [ALT] and aspartate aminotransferase [AST]), which are often discovered incidentally during routine blood tests [24]. These mild elevations are generally reversible with discontinuation or dose reduction of MTX. However, severe liver injury may manifest symptoms such as jaundice, fatigue, abdominal pain, hepatomegaly, and ascites reflecting progressive liver dysfunction. In rare cases, MTX‐LI can lead to liver failure, a life‐threatening condition characterized by the inability of the liver to perform its vital functions [24].

The severity of MTX‐LI is influenced by several risk factors that increase susceptibility to liver damage. Alcohol consumption exacerbates the hepatotoxic effects of MTX by enhancing oxidative stress and inflammation while obesity and T2DM further predispose individuals to liver injury by promoting insulin resistance, and inflammation, all of which contribute to NAFLD or NASH [18]. AIDs, particularly RA and lupus, may also increase the risk of MTX‐induced hepatotoxicity, as the underlying disease or other immunosuppressive treatments may amplify liver damage [19]. Furthermore, genetic factors and cumulative dose of MTX are significant determinants of the severity of MTX‐LI. Chronic MTX therapy, especially in higher doses, increases the risk of developing fibrosis and cirrhosis, conditions that can progress to liver failure over time [25].

## 2. MTX‐LI: Mechanisms and Pathophysiology

### 2.1. Oxidative Stress and Inflammation

MTX primarily enters in the cells via reduced folate carrier (RFC1), with folate receptor (FR)‐mediated endocytosis serving as a secondary route. Intracellularly, folylpolyglutamate synthase (FPGS) catalyzes the addition of polyglutamate residues to MTX, forming MTX polyglutamates (MTX(Glu)_*n*_). These polyglutamated derivatives exhibit enhanced antifolate activity relative to MTX, enabling potent and irreversible inhibition of DHFR. Additionally, MTX polyglutamates inhibit key enzymes including thymidylate synthase (TYMS) and de novo purine synthesis enzymes (GART/ATIC), collectively disrupting nucleotide biosynthesis. This disruption of nucleotide metabolism leads to impaired mitochondrial function, and increased generation of ROS, ultimately contributing to oxidative stress‐mediated cellular damage.

**Figure 1:**
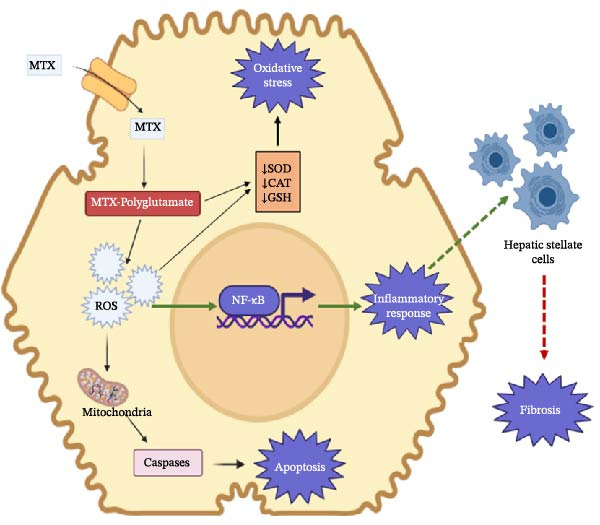
Pathophysiology of methotrexate‐induced liver injury. Methotrexate (MTX) induces injury through oxidative stress, apoptosis, inflammation, and fibrosis in the hepatocytes. Excess ROS triggers mitochondrial dysfunction and apoptosis via the Bax/Bcl‐2/cytochrome‐c pathway. MTX also activates NF‐κB, increasing pro‐inflammatory cytokines (TNF‐α, IL‐1β, IL‐6), while chronic inflammation stimulates hepatic stellate cells thereby promoting fibrosis.

Moreover, the accumulation of intracellular MTX(Glu)_*n*_). ruptures the plasma membrane of hepatocytes, releasing liver enzymes into the bloodstream and triggering oxidative stress, inflammation, steatosis, fibrosis, and apoptosis in hepatocytes [13]. Moreover, MTX‐PG induces oxidative stress in the liver by promoting lipid peroxidation, which releases ROS and inhibits antioxidant response elements as has been depicted in Figure 1. The active metabolite activates various pro‐inflammatory signaling pathways and cytokines, including tumor necrosis factor‐α, nuclear factor kappa B, and interleukins IL‐6, IL‐1β, and IL‐12. Additionally, MTX‐PG depletes hepatic folate levels, reduces RNA and DNA synthesis, and ultimately leads to hepatocytes death [26].

MTX inhibits the enzyme DHFR, which normally converts 7,8‐dihydrobiopterin (BH2) into tetrahydrobiopterin (BH4). This inhibition results in the accumulation of BH2 and a reduction in BH4 levels [27]. Consequently, cellular processes that rely on BH4, such as the function of enzymes like nitric oxide synthetases (NOS), are disrupted. Since these enzymes depend on BH4 for proper functioning, their efficiency is compromised, leading to increased production of ROS as a by‐product [28]. Moreover, because BH4 plays a role in regulating nitric oxide production, which helps mitigate oxidative damage, reduced BH4 levels impair the neutralization of ROS. This imbalance contributes to heightened oxidative stress and an increase in harmful ROS, ultimately causing damage to cells and tissues, including those in the liver [29]. Additionally, in a study conducted by Wang et al., administration of MTX resulted in severe liver damage, alleviated inflammation and overactivated STING signaling thereby demonstrating the role of STING–ERS–ferroptosis axis in triggering MTX‐associated hepatotoxicity [30].

### 2.2. Mitochondrial Dysfunction

As has been discussed, MTX increases the production of ROS in hepatocytes, which causes oxidative damage to mitochondrial membranes, proteins, and DNA, leading to mitochondrial dysfunction. The damage to the mitochondria results in impaired ATP production, which is essential for cell survival and energy metabolism. This mitochondrial dysfunction also leads to the activation of apoptotic pathways, contributing to hepatocyte cell death and eventually liver injury, as is depicted in Figure 1.

The production of ROS is exacerbated by the accumulation of homocysteine, which results from the activation of methylenetetrahydrofolate reductase (MTHFR) by the MTX–polyglutamate (MTX–PGx) complex [31]. ROS triggers an inflammatory response mediated by NF‐κB, leading to mitochondrial depletion and apoptosis activation via the JNK pathway, involving caspase 3 [32]. The cellular defense mechanisms become overwhelmed due to the depletion of several antioxidant agents, including superoxide dismutase (SOD), glutathione (GSH), glutathione peroxidase (GPx), catalase, arginase, and heme oxygenase‐1 (HO‐1), along with the transcriptional suppression of others like nuclear factor erythroid 2‐related factor 2 (Nrf‐2). Apoptosis is further promoted by an imbalance in the regulation of apoptotic factors like BAX and Bcl‐2, which is not compensated for by increased DNA synthesis due to folate depletion [13].

### 2.3. Fibrosis and HSCs Activation

Oxidative stress generated by MTX triggers an inflammatory response, which is mediated by various signaling molecules, including pro‐inflammatory cytokines which in turn attract immune cells like macrophages and neutrophils to the liver, further exacerbating inflammation.

The chronic inflammatory environment induced by MTX leads to the activation of HSCs, which play a pivotal role in the development of liver fibrosis. When activated, HSCs transform into myofibroblast‐like cells that produce excessive extracellular matrix (ECM) components, including collagen, leading to fibrosis as has been depicted in Figure 1 [26]. Additionally, in an in vitro study has shown that molecules accumulating in hepatocytes such as homocysteine and adenosine, when exposed to high doses of MTX, are capable of activating stellate cells and therefore lead to fibrogenesis [26]. This process may progress to cirrhosis if the use of MTX is prolonged, particularly in patients with additional risk factors such as alcohol consumption, obesity, or diabetes.

### 2.4. Altered Drug Metabolism and Enzyme Dysregulation

Genetic polymorphisms may contribute to MTX‐DILI, and thus Eektimmerman et al., conducted a genome‐wide association study (GWAS) using a case–control design to identify genetic risk factors associated with MTX‐DILI in RA patients [33].

Previous studies have identified potential genetic variants linked to MTX‐induced liver toxicity [34]. For example, a large meta‐analysis by Owen et al. demonstrated that the MTHFR C677T polymorphism could be a genetic marker predictive of an increased risk of MTX‐induced liver toxicity [34]. The results demonstrated the involvement of a gene, encoding a protein that may play a role in the mechanism of action of MTX, in exacerbating MTX‐DILI. Specifically, we observed that FTCDNL1, a gene encoding a protein with folic acid transferase and binding activity is expressed predominantly in the brain and liver and the concurrent use of MTX, in combination with FTCDNL1 deficiency, could diminish the protective effects of folic acid, this may contribute to MTX‐DILI [34].

## 3. Phytochemicals in Liver Injury: Mechanisms of Action

Phytochemicals are bioactive compounds naturally occurring in plants that contribute to their color, flavor, and help them develop resist fungi, virus, and insects [35]. These chemical compounds are naturally synthesized by plants through a complex metabolic process and are classified into various categories, such as alkaloids, flavonoids, terpenoids, phenolic acids, and glycosides [35]. Alkaloids, like caffeine and morphine, are known for their effects on the nervous system, while flavonoids, like quercetin and catechins, are recognized for their antioxidant and anti‐inflammatory actions [36]. Conversely, terpenoids, such as curcumin and limonene are involved in plant defense and offer various biological effects, including anti‐cancer properties while both phenolic acids like caffeic acid and ferulic acid, along with glycosides like saponins act as potent antioxidants that modulate immune responses and inflammation [37, 38]. Polyphenols, a broader group that includes flavonoids, phenolic acids, and other compounds like resveratrol, have powerful antioxidant properties that help protect against oxidative stress and inflammatory damage. In the context of liver injury, these natural compounds emerge as promising therapeutic agents by targeting the core pathological processes driving hepatic damage. The hepatoprotective potential of phytochemicals is largely attributed to their multi‐faceted ability to counteract oxidative stress, suppress inflammatory cascades, inhibit stellate cell activation, and directly modulate key cell signaling pathways. This section will systematically explore the role of phytochemicals in mitigating liver injury through four primary mechanisms: their (1) antioxidant, (2) anti‐inflammatory, (3) direct hepatoprotective, and (4) antifibrotic actions, with a specific focus on their modulation of critical molecular targets.

### 3.1. Antioxidant Activities

**Figure 2:**
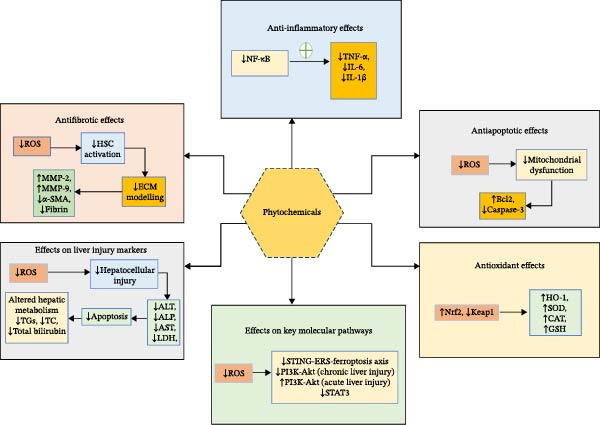
Mechanisms of phytochemical protection against methotrexate (MTX)*–*induced liver injury. MTX triggers oxidative stress, inflammation, dysregulated lipid metabolism, and fibrotic remodeling, which collectively contribute to hepatocellular injury. Phytochemicals counteract these processes through multiple mechanisms: (i) antioxidant activity, by enhancing superoxide dismutase (SOD), catalase (CAT), and Nrf2/ARE signaling; (ii) anti‐inflammatory action, by suppressing NF‐κB activation and downstream cytokines such as TNF‐α and IL‐6; (iii) hepatoprotective effects, by normalizing hepatic lipid metabolism and lowering triglyceride (TG) and total cholesterol (TC) accumulation; (iv) antifibrotic effects, by modulating extracellular matrix turnover and attenuating matrix metalloproteinases (MMP‐2, MMP‐9); and (v) regulation of key molecular pathways, including JAK–STAT and PI3K–Akt signaling. Together, these coordinated actions reduce MTX‐induced hepatotoxicity and promote overall hepatoprotection.

Hepatocytes are highly vulnerable to oxidative stress due to their central role in metabolic processes and detoxification. The liver is exposed to a variety of ROS from both endogenous metabolic activity and exogenous insults, including drugs like MTX. Antioxidants in hepatocytes work through enzymatic systems, such as SOD, GPx, and catalase, which neutralize ROS, as well as non‐enzymatic systems like GSH and vitamin E. These antioxidants help protect liver cells from oxidative damage, apoptosis, and necrosis, which can otherwise lead to liver injury and fibrosis. Phytochemicals, including flavonoids, such as quercetin and catechins, and phenolic acids, such as resveratrol and curcumin, have been shown to exhibit powerful antioxidant properties. They act by scavenging ROS, enhancing the activity of antioxidant enzymes, and reducing the expression of oxidative stress markers. Flavonoids, through their ability to regulate redox‐sensitive transcription factors like Nrf2, promote the expression of antioxidant enzymes and protect hepatocytes from damage as depicted in Figure 2. By mitigating oxidative stress, these phytochemicals play a crucial role in preventing the liver damage caused by drugs like MTX.

### 3.2. Anti‐Inflammatory Effects

Phytochemicals derived from medicinal plants are crucial in managing various inflammatory disorders [39]. For instance, alkaloid compounds like hyoscine from *Datura stramonium* and berberine from *Berberis vulgaris* have been recognized as licensed anti‐inflammatory agents [40]. The extracts of medicinal plants are widely suggested as alternative treatments as well as template for drug discovery for inflammatory disorders [41]. Despite limited information on the secondary metabolites of these plants, numerous studies indicate that people in developing countries primarily rely on medicinal herbs for treating inflammatory conditions [42]. Phytochemicals which are chemically secondary metabolites synthesized in plant parts have been shown to effectively address a wide range of inflammatory diseases. Additionally, natural products from foods, herbs, and plants are actively being explored in clinical and laboratory settings for their anti‐inflammatory properties and potential health benefits [43, 44].

### 3.3. Hepatoprotective Mechanisms

The liver has a remarkable ability to regenerate after injury, but this process can be impaired by sustained damage. In a study conducted by Seo et al., phytochemicals like naringin help mitigate the harmful effects of ethanol consumption by enhancing the metabolism of ethanol and lipid and boosting the antioxidant defense system of liver, resulting in a significant reduction in the levels of triglycerides (TGs) and total cholesterol (TC) in both the liver and plasma [45]. Phytochemicals can modulate both apoptosis (programed cell death) and autophagy (cellular degradation and recycling) pathways and can therefore minimize liver damage and promote cell survival despite repetitive toxic insults, offering potential adjunctive treatments to MTX‐LI.

### 3.4. Fibrosis Inhibition

Matrix metalloproteinases (MMPs) play a key role in ECM breakdown during liver fibrosis, with their activity varying at different disease stages. MMP‐1 helps slow progression by degrading fibrillar ECM, while activated HSCs increase MMP‐2, ‐9, and ‐13 expression in animal models, potentially either exacerbating or mitigating fibrosis depending on the model and stage. In early fibrosis, elevated MMP levels can damage tissue and promote HSC activation, worsening fibrosis, while reduced MMP activity in later stages facilitates ECM buildup and disease progression [46]. Several preclinical studies have investigated the use of phytochemicals to target MMPs and related signaling pathways in preventing the progression of liver fibrosis and its associated co‐morbidities [47]. Phytochemicals like apigenin (API) and naringenin upregulate MMP‐2 to counter fibrosis while polyphenols like chlorogenic acid and isoorientin upregulate MMP‐9 to resolve hepatic fibrosis [46]. These compounds offer potential therapeutic benefits in preventing or slowing the progression of fibrosis induced by drugs like MTX as depicted in Figure 2.

### 3.5. Molecular Targets

Phytochemicals, bioactive compounds derived from plants, are recognized for their therapeutic potential and provides leads for drug and nutraceutical development. This study highlights their role in influencing key signaling pathways and biological processes. For example, curcumin from turmeric, resveratrol from grapes, and epigallocatechin gallate (EGCG) from green tea modulate essential pathways like PI3K/AKT, MAPK‐ERK, Wnt, and Hedgehog, which play crucial roles in cellular functions and disease progression [48]. Curcumin exhibits anticancer effects by inhibiting cell proliferation, promoting apoptosis, and blocking angiogenesis [49]. Resveratrol offers anti‐inflammatory benefits by targeting cytokine production and oxidative stress pathways, helping manage chronic inflammation linked to various diseases [50]. The antioxidant properties of EGCG neutralize free radicals, reducing oxidative damage and promoting cellular health [51].

Furthermore, phytochemicals also have potential to regulate pathways in metabolic disorders like diabetes and obesity by regulating glucose metabolism, lipid profiles, and insulin sensitivity. In neurodegenerative diseases, compounds like berberine and ginsenosides from ginseng show promise in improving insulin sensitivity, protecting neurons, and reducing neuroinflammation and oxidative stress, enhancing cognitive function and overall brain health [52]. Wang et al. highlighted the crucial involvement of the STING‐ERS‐ferroptosis axis in mediating MTX‐induced hepatotoxicity as depicted in Figure 2, which causes significant liver damage, inflammation, and lipid peroxidation, suggesting that targeting this axis could offer a promising therapeutic strategy for treating hepatotoxicity [30].

## 4. Phytochemicals in MTX‐LI

**Figure 3:**
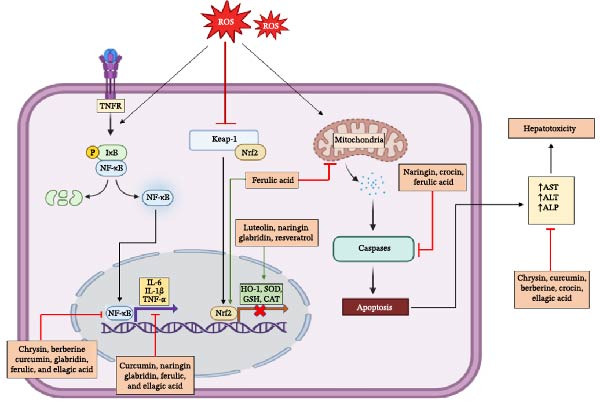
Mechanisms of phytochemical‐mediated protection against methotrexate (MTX)‐induced‐hepatotoxicity: This figure summarizes the protective role of phytochemicals in hepatotoxicity, highlighting their ability to modulate key molecular pathways. Phytochemicals attenuate inflammation by suppressing pro‐inflammatory cytokines (e.g., TNF‐α, IL‐6) and inhibiting NF‐κB activation. They regulate apoptosis by modulating Bcl‐2 family proteins and caspase activity, while enhancing antioxidant defenses through Nrf2‐mediated upregulation of enzymes like SOD and GSH.

Phytochemicals are increasingly being studied for their potential to mitigate liver injury, including that caused by drugs like MTX, since phytochemicals can modulate oxidative stress, inflammation, and fibrosis, which are central mechanisms in liver injury, as has been summarized in Figure 3. For instance, compounds like silymarin (from milk thistle) and curcumin have demonstrated the ability to protect liver cells from drug‐induced toxicity and prevent the progression of liver damage [53]. Investigating these natural compounds as adjunct therapies or nutraceuticals could offer complementary alternatives to conventional MTX‐based treatments, potentially enhancing therapeutic outcomes while minimizing adverse effects.

### 4.1. Flavonoids

#### 4.1.1. Apigenin

Apigenin ‐ (4′, 5,7‐trihydroxyflavone), a potent antioxidant and anti‐inflammatory agent, belongs to a class of polyphenols known as flavonoids and is found abundantly in fruits, vegetables, and herbs such as parsley, celery, chamomile, and green pepper [54]. Aigenin is biosynthetically derived from L‐phenylalanine or L‐tyrosine through the phenylpropanoid pathway and flavone synthesis pathway [55]. In a study conducted by Sahindokuyucu‐Kocasari et al. [56], the therapeutic dose and treatment regime of apigenin were showed indifferent groups of male mice. Following intraperitonal administration at a dose of 3 mg/kg. To evaluate its protective effect against a single intraperitoneal dose of MTX (20 mg/kg) given on day 4, the MTX + apigenin group received apigenin pretreatment for 7 days, with MTX co‐administered on the fourth day. Control groups received either saline or the single MTX dose alone [56]. Kocasari et al. demonstrated the ameliorative effects of apigenin on MTX‐LI by attenuating oxidative stress and apoptosis, inflammation and tissue injury [56].

#### 4.1.2. Chrysin

Chrysin (5,7‐dihydroxyflavone), a dihydroxyflavone, is naturally found in honey, propolis, and passion flowers (*Passiflora caerulea* and *Passiflora incarnata*) [57]. Notably, a study by Ali et al. examined its hepatoprotective effects in a rat model of MTX‐LI and demonstrated its therapeutic potential in mitigating liver damage by attenuating oxidative stress, inflammation, and apoptosis of hepatocytes [58]. In their investigation, chrysin was administered orally at two doses (40 and 80 mg/kg) for 20 days to evaluate its protective effect. A single dose of MTX (20 mg/kg, ip) was co‐administered on the 18th day to induce hepatotoxicity, with assessments performed on day 21 [58].

#### 4.1.3. Diosmin

Diosmin (3′, 5,7‐trihydroxy‐4′‐methoxyflavone‐7‐rhamnoglucoside), a flavone glycoside, is found most commonly in citrus plants and was first isolated from *Scrophularia nodosa* L. in 1925 [59]. It has been since used in the treatment of hemorrhoids, varicose veins, and other circulatory issues. The flavonoid plays a multifaceted role as an anti‐inflammatory agent while alleviating endothelial cell activation and leukocyte adhesion [60]. In a study conducted by Abdel‐Daim et al., diosmin (50–100 mg/kg) for a duration of 10 days, was found to significantly reduce the levels of malondialdehyde (MDA) and nitric oxide and increase the levels of GSH, SOD, and catalase thereby ameliorating the hepatic injury induced by MTX in mice [61].

#### 4.1.4. Epicatechin

Epicatechin, ((2R, 3S)‐2‐(3,4‐Dihydroxyphenyl)‐3,4‐dihydro‐2H‐chromene‐3,5,7‐triol), a flavan‐3‐ol, is a major chemical constituent of traditional herbal remedies like the *Uncaria rhynhophylla* and can also be abundantly found in cocoa and tea as well as in grapes (*Vitis vinifera*) [62]. Azadnasab et al. demonstrated that the administration of epicatechin (100 mg/kg) for a duration of 10 days reduced liver dysfunction by enhancing the antioxidant defense system, anti‐inflammatory effects, and alleviation of histopathological damage in MTX hepatotoxicity in mice [63].

#### 4.1.5. Galangin

Galangin (3,5,7‐trihydroxyflavone), a flavonol, is abundantly found in *Alpinia galanga* (galangal) and propolis and isolated from the roots of plants belonging to *Zingiberaceae* family [64]. The trihydroxyflavone plays an important role in diseases like neurodegenerative diseases, RA, osteoarthritis, dermatological conditions and cancer. In a study conducted by Manal, Galangin was found to possess a hepatoprotective effect mediated by attenuating oxidative damage, inflammation, and apoptosis in rats modeling MTX ‐induced hepatic injury [65]. The therapeutic regimen involved administering Galagin for 10 days, with a single dose of MTX (20 mg/kg) given on day 7 [65].

#### 4.1.6. Glabridin

Glabridin, ((3*R*)‐6′′, 6′′‐Dimethyl‐6′′*H*‐pyrano [2′′, 3′′:7,8]isoflavan‐2′, 4′‐diol), a prenylated isoflavone, extracted from the root extracts of licorice plant (*Glycyrrhiza glabra*), is found to be a potent anti‐inflammatory, antioxidant, antimicrobial, antineoplastic agent while being a strong inhibitor of platelet activation [66]. It is also considered to be a potent activator of peroxisome proliferator‐activated receptor γ (PPAR‐γ) while being an antagonist of tyrosinase, P‐glycoprotein and CYP2E1 [67]. The isoflavanoid when administered orally for 11 consecutive days, at a dose of 10–40 mg/kg, ameliorated hepatotoxicity induced by MTX in mice by upregulating the expression of Nrf2 and BAX while inactivating NF‐κB [68].

#### 4.1.7. Gossypin

Gossypin (2‐(3,4‐dihydroxyphenyl)‐3,5,7‐trihydroxy‐4‐oxo‐4H‐chromen‐8‐yl b‐D‐gluco‐pyranoside), a flavonoid glycoside, is originally isolated from flowers and roots of Malvaceae family, namely *Hibiscus vitifolius*, *Hibiscus esculentus*, and *Gossypium indicum* [69]. In a study conducted by Mohamed et al., oral administration of gossypin (10 mg/kg) for 10 days was found to restore the normal architecture of hepatocytes and expression of TGF‐β, BAX, caspases and NF‐κB while significantly downregulating the expression of P‐ glycoprotein (P‐gp) in hepatotoxicity induced by MTX in rats [70].

#### 4.1.8. Hesperidin

Hesperidin ((2*S*)‐3′, 5‐Dihydroxy‐4′‐methoxy‐7‐[α‐L‐rhamnopyranosyl‐(1→6)‐β‐D‐glucopyranosyloxy]flavan‐4‐one), a flavone, belonging to the class of flavone compounds, is primarily found in citrus fruits like grapefruits (*Citrus paradise*), oranges (*Citrus sinensis*), tangerines (*Citrus reticulata*), and lemons (*Citrus limon*) [71]. The flavonoid demonstrates a very potent antioxidant property by reducing the production of ROS and increasing the activity of antioxidant enzymes like catalase and SOD [71]. Abdelaziz et al. demonstrated the hepatoprotective role by hesperidin (50 mg/kg) by upregulating Nrf2/HO‐1/Bcl2 signaling and downregulation of NF‐κB in rats. The treatment regime consisted of oral administration of Hesperidin at a dose of 50 mg/kg/day for 28 days, with a single intraperitoneal injection of MTX (20 mg/kg) administered on the 28th day [72].

#### 4.1.9. Luteolin

Luteolin (3′, 4′, 5,7‐Tetrahydroxyflavone), a flavonoid, present primarily in various fruits and vegetables like carrot, cabbages, onions and broccoli is isolated usually from a flowering plant in Lamiaceae family known as *Salvia tomentosa* [73]. In a study conducted by Dar et al., the hepatoprotective effects of Luteolin were investigated in rat model of MTX induced hepatotoxicity using a regimen where luteolin (50 mg/kg) was administered orally for 14 days to male rats, and challenged with a single dose of MTX (20 mg/kg, i.p.) on day 9 [74]. Luteolin was found to stimulate Nrf2 signal transduction which triggered the antioxidant response while downregulating the NF‐κB expression that demonstrated the anti‐inflammatory and the anti‐apoptotic response in the MTX‐ induced hepatotoxicity in rats [74].

#### 4.1.10. Morin

Morin (3,5,7,2′, 4′‐pentahydroxyflavone), a dihydroxyflavone, is primarily a major constituent of various plants from the *Moraceae* (mulberry) family. Morin is widely recognized for its wide range of antioxidant, anti‐inflammatory, antidiabetic, antineoplastic, and antimicrobial properties [75]. In a rat model of hepatotoxicity induced by a single intraperitoneal injection of MTX (20 mg/kg) on day 5, Kızıl et al. demonstrated that pretreatment with morin (50 or 100 mg/kg, orally) for 10 days mitigated oxidative stress and apoptosis [76]. Kızıl et al. demonstrated that treatment with morin mitigated oxidative stress and apoptosis in the MTX‐induced hepatic injury model in rats. Morin also restored the normal physiological levels of ALP, ALT, and AST in liver tissue samples while downregulating the expression of matrix metalloproteinases; MMP‐2 and MMP‐9 and MAPK downstream signaling [76].

#### 4.1.11. Naringin and Naringenin

Naringin ((2*S*)‐4′, 5‐dihydroxy‐7‐[α‐L‐rhamnopyranosyl‐(1→2)‐β‐D‐glucopyranosyloxy]flavan‐4‐one), a flavanone‐7‐O‐glycoside, occurs naturally in the citrus fruits like the grapefruits and is responsible for the bitter taste in these fruits [77]. The aglycol form of naringin, known as naringenin [(2*S*)‐4′, 5,7‐Trihydroxyflavan‐4‐one] is produced by the hydrolytic actions of the liver enzyme naringenase on naringin [78]. In a study conducted by Elsawy et al., Naringen (20–80 mg/kg) was administered orally for 10 days with a single dose of MTX (20 mg/kg, i.p.) on day 4 [79]. However, Naringenin was administered at a dose of (50 mg/kg, orally) for 10 days with a single MTX challenge (20 mg/kg, i.p.) on day 9 [80]. Both of these flavanones have demonstrated significant hepatoprotective effects by reversing hepatic injury induced by MTX in rats by restoring the levels of antioxidants enzymes, SOD, and catalase, while significantly reducing MDA and nitric oxide [79, 80]. They also countered the increase in pro‐inflammatory cytokines like IL‐6 and TNF‐α, thereby mediating the resolution of inflammation [79]. However, both flavanones may however cause drug–drug interaction and may lead to hepatotoxicity since they inhibit CYP3A4.

#### 4.1.12. Phloridzin

Phloridzin (1‐[2,4‐dihydroxy‐6‐[(2S,3R,4S,5S,6R)‐3,4,5‐trihydroxy‐6‐(hydroxymethyl) oxan‐2‐yl]oxyphenyl]‐3‐(4‐hydroxyphenyl) propan‐1‐one), a dihydrochalcone, was first isolated from the bark of apple trees and is mainly distributed in the plants of the *Malus* genus [81]. The flavonoid glycoside mainly found in apples and strawberries is a potent antioxidant, anti‐inflammatory and competitive inhibitor of glucose transporters and platelet activation [82]. In a study conducted by Khalifa et al., phloridzin was demonstrated to have ameliorative effects on MTX‐induced hepatic injury in rats. Using a dosing regimen where phloridzin (40 mg/kg/day, p.o.) was administered for 10 consecutive days with an MTX challenge (20 mg/kg, i.p.) on day 3, the study showed that the flavonoid worked by downregulating the expression of NF‐κB and MAPK while activating nuclear factor erythroid (NrF2) and c‐Jun‐terminal Kinase (JNK), thereby leading to the induction of heme oxygenase‐1 (HO‐1) which provides a potent anti‐apoptotic function [82].

#### 4.1.13. Rutin

Rutin (3′, 4′,5,7‐tetrahydroxy‐3[α‐L‐rhamnopyranosyl‐(1→6)‐β‐D‐glucopyranosyloxy]flavone), also known as rutoside, quercetin‐3‐O‐rutinoside or sophorin, is a flavonol quercetin is usually found in plant species like *Carpobrotus edulis* and *Ruta graveolens* and other citrus plants [83]. The dietary flavanol has also been found to alleviate conditions related to vascular dysfunctions such as epithelial dysfunctions [84]. A study conducted by Erdogan et al., found Rutin to be a promising adjuvant against MTX‐LI, as it significantly mitigated oxidative stress by reducing MDA levels and upregulating GSH, CAT, and SOD, in addition to demonstrating marked histological improvement. In the experiment, rats that received a single MTX dose (20 mg/kg, i.p.) were treated with rutin (100 mg/kg, i.p.) for the 10 consecutive days, which successfully mitigated the liver damage [85].

### 4.2. Terpenoids

#### 4.2.1. Lycopene

Lycopene (ψ,ψ‐Carotene), a carotenoid and one of the most potent antioxidants in the terpenoid family, is primarily found in tomatoes, watermelon, and pink grapefruits and is fat soluble in nature [86]. As a tetraterpene, lycopene is renowned for its free radical‐scavenging properties and its role in preventing oxidative stress‐related diseases while being extensively studied for its anti‐inflammatory, anticancer, and cardioprotective effects. Furthermore, in a study conducted by Yucel et al., lycopene (10 mg/kg/day) modulated pro‐inflammatory cytokines, specifically reducing the levels of TNF‐α and IL‐1β, thereby mitigating liver inflammation and attenuated MTX ‐induced hepatotoxicity, emphasizing its antioxidant and anti‐inflammatory properties that has potential to protect liver from MTX associated damage [87].

#### 4.2.2. Carvacrol

Carvacrol (5‐isopropyl‐2‐methylphenol), a monoterpenoid phenol, is primarily found in the essential oils of *Origanum vulgare* (oregano) and *Thymus vulgaris* (thyme) and exerts potent antioxidant effects by scavenging ROS and enhancing endogenous antioxidant enzymes, while also regulating inflammatory pathways by inhibiting pro‐inflammatory cytokines [88]. In a study by Bozkurt et al., a single dose of carvacrol (73 mg/kg) significantly reduced MDA levels, elevated total antioxidant capacity (TAS), and reduced liver damage, indicating its hepatoprotective effects against MTX‐LI [89].

#### 4.2.3. β‐Carotene

β‐carotene (β‐Carotene), a carotenoid primarily found in carrots, sweet potatoes, and leafy greens, and also from certain algae like *Dunaliella salina* is a potent antioxidant and free radical scavenger which enhances cellular antioxidant defenses, and protect against oxidative stress and inflammation [90]. In a study by Vardi et al., β‐carotene demonstrated a protective effect against MTX‐induced hepatic injury. The study showed that in rats pretreated with β‐carotene (10 mg/kg/day, i.p.) for 24 days prior to an MTX challenge (20 mg/kg, i.p.) on day 21, the treatment significantly decreased AST and ALT levels, with other parameters similar to the control group in rats, indicating its protective effect against MTX‐induced oxidative injury of liver [91].

#### 4.2.4. Astaxanthin

Astaxanthin ((3*S*,3′*S*)−3,3′‐Dihydroxy‐β,β‐carotene‐4,4′‐dione), a keto‐carotenoid, a metabolite of zeaxanthin and canthaxanthin, produced naturally in freshwater microalgae *Haematococcus pluvialis*, and the yeast fungus *Xanthophyllomyces dendrorhous*. It is also found in animals like salmon, shrimp, and flamingos, get the red‐orange pigmentation being fed on astaxanthin‐producing organisms [92]. In a study by Azadian et al., showed that treatment with astaxanthin (7.5–15 mg/kg) significantly reduced blood levels of ALT, AST, ALP, and LDH, as well as oxidative stress markers, while markedly increased the activity of antioxidant enzymes and the expression of Nrf2 and HO‐1 genes in liver tissue. Astaxanthin administration improved histopathological lesions and oxidative and inflammatory changes, suggestive of its potential in mitigating MTX‐induced hepatotoxicity [93].

#### 4.2.5. 18β‐Glycyrrhetinic Acid

18β‐glycyrrhetinic acid (3β‐hydroxy‐11‐oxo‐18β, 20β‐olean‐12‐en‐29‐oic‐acid), key triterpenoid saponin glycoside metabolite of glycyrrhizic acid, after being metabolized by glucuronidase in plants and by intestinal bacteria after oral ingestion [94]. The terpenoid and its derivatives demonstrate a wide range of biological and pharmacological activities, such as antitumor, anti‐inflammatory, antioxidant, antiviral, antimicrobial, antidiabetic, hepatoprotective, cardioprotective, and neuroprotective effects [95–97]. The protective effect of 18β‐GA against MTX‐LI was demonstrated using two distinct regimens: a 7‐day oral pretreatment (50 or 100 mg/kg) and a 7‐day oral post‐treatment. Both schedules, in response to a single MTX challenge (20 mg/kg, i.p.), were effective in reducing liver enzymes, bilirubin, pro‐inflammatory cytokines, and oxidative stress, while also improving liver histology, modulating Bax/Bcl‐2 expression, and activating Nrf2 and PPARγ [98].

#### 4.2.6. Crocin

Crocin (Bis[β‐D‐glucopyranosyl‐(1→6)‐β‐D‐glucopyranosyl] 8,8′‐diapocarotene‐8,8′‐dioate), a carotenoid compound found in *Crocus sativus* (saffron), is known for its potent antioxidant and anti‐inflammatory properties [99]. In a study, pretreatment with crocin (25–50 mg/kg) for a duration of 10 days, significantly alleviated MTX‐LI by increasing antioxidant defense, restoring GSH content, and enhancing catalase, and SOD activity [100]. Additionally, crocin reduced oxidative stress markers, including TNF‐α, IL‐1β, lipid peroxidation, and nitric oxide levels, suggesting its protective role in mitigating liver injury caused by MTX [100].

### 4.3. Phenolic Compounds

#### 4.3.1. Ferulic Acid

Ferulic acid ((2*E*)‐3‐(4‐hydroxy‐3‐methoxyphenyl)prop‐2‐enoic acid), a phenolic phytochemical commonly found in grains, fruits, and vegetables, is recognized for its antioxidant, anti‐inflammatory, and hepatoprotective effects [101]. In a study conducted by Mahmoud et al. [102], ferulic acid (25–50 mg/kg) prevented histological alterations and improved liver function markers in MTX‐induced hepatotoxicity. Ferulic acid suppressed oxidative stress, reduced serum TNF‐α and IL‐1β, and decreased hepatic NF‐κB p65, Bax, and caspase‐3, while enhancing Bcl‐2, Nrf2, NQO1, HO‐1, and PPARγ expressions. These findings suggest that ferulic acid protects against MTX‐LI by activating PPARγ, Nrf2/HO‐1 signaling, and mitigating oxidative stress, inflammation, and cell death [102].

#### 4.3.2. Sinapic Acid

Sinapic acid ((2*E*)‐3‐(4‐Hydroxy‐3,5‐dimethoxyphenyl)prop‐2‐enoic acid), a phenolic acid found in mustard seeds, oats, and various fruits, is known for its antioxidant, anti‐inflammatory, and hepatoprotective properties [103]. In a study conducted by Ahmad et al., sinapic acid (20 mg/kg) pretreatment for a duration of 10 days significantly restored liver function indices, such as ALT, AST, and ALP, which were altered by MTX exposure while improving antioxidant defense mechanisms (GSH, SOD, CAT) and reducing oxidative stress markers (MDA, NO) and inflammatory cytokines (TNF‐α, IL‐1β, MPO). Sinapic acid mitigated MTX‐induced hepatic damage by inhibiting apoptosis, stimulating Nrf2/HO‐1‐mediated antioxidant enzymes, and inhibiting NF‐κB signaling, as confirmed by histopathological analysis showed hepatoprotective activities [104].

#### 4.3.3. Chlorogenic Acid

Chlorogenic acid ((1*S*,3*R*,4*R*,5*R*)‐3‐{[(2*E*)‐3‐(3,4‐Dihydroxyphenyl)prop‐2‐enoyl]oxy}‐1,4,5‐trihydroxycyclohexane‐1‐carboxylic acid), chemically 5‐caffeoylquinic acid, is an ester of caffeic and quinic acids found in coffee, fruits, and vegetables, and is a potent antioxidant, anti‐inflammatory, and neuroprotective agent that scavenges free radicals, reduces oxidative stress, and modulates inflammatory pathways [105]. In a study conducted by Ali et al. [106], chlorogenic acid pretreatment (50–100 mg/kg) alleviated MTX‐induced hepatic injury by decreasing oxidative stress, inhibiting pro‐inflammatory and apoptotic mediators (COX‐2, iNOS, Bax, Bcl‐2, Caspases 3 and 9), and enhancing antioxidant defenses, demonstrating its hepatoprotective potential [105].

#### 4.3.4. Chicoric Acid

Chicoric acid ((2*R*,3*R*)‐2,3‐Bis{[(2*E*)‐3‐(3,4‐dihydroxyphenyl)prop‐2‐enoyl]oxy})butanedioic acid, a caffeoyltartaric acid derivative found in Echinacea species, is recognized for its antioxidant, anti‐inflammatory, and hepatoprotective properties [107]. In a study conducted by Hussein et al. [108], chicoric acid (25–50 mg/kg) effectively suppressed ROS and lipid peroxidation while enhancing antioxidant defenses in MTX‐treated rats. Furthermore, chicoric acid upregulated hepatic nuclear factor erythroid 2‐related factor 2 (Nrf2), heme oxygenase‐1 (HO‐1), NAD(P)H quinone dehydrogenase 1 (NQO‐1), and peroxisome proliferator‐activated receptor gamma (PPARγ), attenuating inflammation. Additionally, chicoric acid inhibited apoptosis by increasing Bcl‐2 expression and reducing Bax, cytochrome c, and caspase‐3 levels. These findings suggest that chicoric acid protects against MTX‐induced oxidative stress, inflammation, and liver injury by activating Nrf2/HO‐1 signaling and PPARγ, making it a promising agent for liver protection [108].

#### 4.3.5. Ellagic Acid

Ellagic acid (2,3,7,8‐Tetrahydroxy[1]benzopyrano[5,4,3‐*cde*][1]benzopyran‐5,10‐dione), a polyphenolic compound found in various fruits, such as pomegranates, strawberries, and raspberries, is known for its antioxidant, anti‐inflammatory, and hepatoprotective properties. Ellagic acid has been shown to significantly prevent oxidative stress, mitochondrial dysfunction, apoptosis, and inflammation induced by MTX [109]. In a study conducted by Ebrahimi et al. [110], ellagic acid (5–10 mg/kg) treatment resulted in the upregulation of nuclear factor erythroid 2‐related factor 2 (Nrf2) and heme oxygenase‐1 (HO‐1), both of which were downregulated in MTX‐treated rats, while also inhibiting the NF‐κB signaling pathway. Furthermore, ellagic acid pre‐treatment notably reduced the activities of aminotransferases and alkaline phosphatase (ALP), improved oxidative stress markers, and enhanced antioxidant enzyme activities [111]. Histological examination confirmed the remarkable hepatoprotective effect of ellagic acid, demonstrating its potential as a therapeutic agent against MTX‐induced hepatotoxicity.

### 4.4. Alkaloids

#### 4.4.1. Berberine

Berberine (9,10‐dimethoxy‐7,8,13,13a‐tetradehydro‐2′*H*‐[1,3]dioxolo[4′, 5′:2,3]berbin‐7‐ium), an alkaloid found in *Berberis* species, exhibits antioxidant, anti‐inflammatory, and hepatoprotective effects [112]. In a study conducted by Mahmoud et al., in a rat model of MTX‐induced hepatotoxicity, berberine was shown to improve body weight, liver function markers, and oxidative stress parameters, including TNF‐α, lipid peroxidation, and caspase‐3, while enhancing serum albumin and liver antioxidant defenses. This protective effect was demonstrated using a regimen where berberine (25 or 50 mg/kg/day, orally) was administered for 7 days in relation to a single MTX challenge (20 mg/kg, i.p.), confirming its efficacy as both a pre‐treatment and a post‐treatment [98]. Histological analysis revealed improved liver structure and reduced Bax expression, with upregulation of Nrf2, HO‐1, and PPARγ, indicating protective effect of berebrine through activation of the Nrf2/HO‐1 pathway and PPARγ. These findings suggest that bereberine mitigates MTX‐LI by reducing oxidative stress, apoptosis and inflammation [98].

### 4.5. Polyphenols

#### 4.5.1. Resveratrol

Resveratrol (5‐[(*E*)‐2‐(4‐Hydroxyphenyl)ethen‐1‐yl]benzene‐1,3‐diol), a polyphenolic compound found in grapes and berries, is well‐known for its antioxidant, anti‐inflammatory, and hepatoprotective properties [113]. In a rat model of MTX‐induced hepatotoxicity, resveratrol (20 mg/kg/day, i.p.) administered for 3 days prior to and during MTX exposure (7 mg/kg/day, i.p., for 3 days) significantly reduced liver injury markers (AST, ALT, ALP) and improved histological outcomes compared with MTX alone [114, 115]. It also decreased hepatic MDA, myeloperoxidase, and collagen levels while increasing GSH, suggesting its ability to inhibit oxidative stress and lipid peroxidation. Furthermore, resveratrol treatment attenuated elevated TNF‐α levels, liver enzyme activities, and oxidative stress markers, highlighting its protective effect against MTX‐induced liver damage as has been depicted in Figure 3. These findings suggest that resveratrol is a promising therapeutic strategy for preventing MTX‐induced hepatotoxicity through its antioxidant and anti‐inflammatory properties [114].

#### 4.5.2. Punicalagin

Punicalagin (2,3‐(S)‐hexahydroxydiphenoyl‐4,6‐(S, S)‐gallagyl‐D‐glucose), a major polyphenolic compound found in pomegranate (*Punica granatum*), is recognized for its potent antioxidant and anti‐inflammatory properties [116]. In a study on MTX‐induced hepatotoxicity in mice, oral administration of punicalagin (25 or 50 mg/kg/day) for 10 days prior to a single intraperitoneal MTX challenge (20 mg/kg) significantly reduced serum transaminases, ALP, and LDH levels, as well as hepatic oxidative stress markers, while enhancing antioxidant defenses in the liver [117]. Punicalagin treatment also attenuated the increase in NF‐κB p65 expression, pro‐inflammatory cytokines (IL‐6, TNF‐α), and pro‐apoptotic proteins (caspase‐3, Bax), while upregulating Bcl‐2 and Nrf2 expressions. These findings suggest that punicalagin mitigates oxidative stress, inflammation, and apoptosis, offering potential therapeutic benefits for preventing MTX‐LI [117].

#### 4.5.3. Curcumin

Curcumin (1*E*,6*E*)‐1,7‐Bis(4‐hydroxy‐3‐methoxyphenyl)hepta‐1,6‐diene‐3,5‐dione), a polyphenolic compound found in turmeric, is well‐known for its antioxidant, anti‐inflammatory, and hepatoprotective properties [118]. In a study by Hemeida et al., curcumin (100 mg/kg, i.p.) administered once daily for 5 days following MTX challenge markedly ameliorated MTX‐induced hepatotoxicity, as evidenced by improved biochemical parameters and attenuation of histological alterations, including fatty acid metabolism, necrosis, and inflammation in hepatocytes and sinusoidal lining cells [119]. Biochemically, curcumin restores elevated serum ALT and AST activities, increased liver antioxidant enzyme levels (SOD, CAT) and reduced GSH, while decreasing lipid peroxidation (MDA levels) as summarized in Figure 3. These findings suggest that curcumin protects against MTX‐induced liver damage by restoring the oxidant/antioxidant balance [119].

#### 4.5.4. Mangiferin

Mangiferin (2‐(β‐D‐Glucopyranosyl)‐1,3,6,7‐tetrahydroxy‐9*H*‐xanthen‐9‐one), a xanthone glycoside primarily found in mangoes (*Mangifera indica*), is recognized for its antioxidant, anti‐inflammatory, and hepatoprotective effects [120]. In a study on MTX‐LI in rats, pretreatment with mangiferin (50 or 100 mg/kg, i.p.) for 10 days, with an MTX challenge (40 mg/kg, i.p.) on the seventh day, significantly restored hepatic architecture and function by attenuating oxidative stress through a reduction in MDA and an increase in reduced GSH and Nrf2 expression [121]. Additionally, mangiferin suppressed inflammation by downregulating the NF‐κB/NLRP3 inflammasome pathway and exhibited anti‐fibrogenic potential by reducing fibrous tissue deposition and hepatic α‐SMA expression. These results highlight mangiferin’s potential as a therapeutic agent against MTX‐induced hepatotoxicity [121].

### 4.6. Others

These studies explore the broader role of plant‐based remedies, providing insight into their hepatoprotective properties. Although not always centered on individual phytochemicals, these plant extracts show promise in enhancing liver function and reducing oxidative stress, offering a valuable addition in supporting liver health in the context of MTX toxicity. Most of these studies listed in Table 1 were conducted on rodent models; however, one notable study conducted by Hagag et al., investigated the hepatoprotective potential of black seed oil in young patients undergoing treatment for Acute lymphoid leukemia and demonstrating signs of hepatic injury by MTX [136]. Preliminary evidence from this study suggests the supplementation of *Nigella sativa* (black cumin) mitigates MTX‐induced hepatotoxicity and enhances survival rates in pediatric ALL patients, supporting its potential as an adjunctive therapy during MTX treatment regimens [136].

**Table 1:** Role of medicinal plants extracts in countering methotrexate‐induced liver injury.

Plant extract	Dose of methotrexate and animal	Treatment regimen	Duration of treatment	Parameters and markers evaluated	References
*Oryza sativa L. indica*	20 mg/kg/b.w to rats	Nanocomposites (10–20 mg/kg/b.w)	10 days	↓ TBA, ↓ALT, ↓ALP, ↓LDH ↑SOD ↑GSH	[122]
*Chamaecyparis lawsoniana*	20 mg/kg/b.w to rats	Ethanolic extract of aerial parts (200–400 mg/kg/b.w)	10 days	↓IL6, ↓TNF‐α ↓IL‐1β	[123]
*Crocus sativus*	20 mg/kg/b.w to rats	Ethanolic extract of flowers (80 mg/kg/b.w)	10 days	↓ALT, ↓ALP, ↓LDH, ↓MDA ↓NO, ↑SOD	[124]
*Allium sativum*	20 mg/kg/b.w to rats	Diallyl sulfide from the plant (50 mg/kg/day)	10 days	↓ALT, ↓ALP, ↓LDH, ↓NO, ↑Nrf‐2, ↓Keap‐1 ↓NF‐κB	[125]
*Bohadschia marmorata*	20 mg/kg/b.w to mice	Ethanolic extract of homogenized tissue (30 mg/kg/b.w)	10 days	↑GSH ↑SOD, ↓CAT, ↓MDA	[126]
*Gingko biloba*	20 mg/kg/b.w to rats	Ethanolic extract of leaf extract (60 mg/kg/day)	10 days	↓ALT, ↓ALP, ↓AST, ↓IL6, ↓p‐STAT3	[127]
*Glycyrrhiza glabra*	20 mg/kg/b.w	100–400 mg/kg/b.w	15 days	↓ALT, ↓ALP, ↓AST, ↑GSH ↑SOD, ↓CAT, ↓TNF‐α, ↓IL6, ↓IL‐1β	[128]
*Vitis vinifera*	20 mg/kg/b.w to rats	Ethanolic extract of dried seeds (100 mg/kg/b.w)	15 days	↑GSH ↑SOD, ↓CAT, ↓MDA	[129]
*Hibiscus sabdariffa*	20 mg/kg/b.w to rats	Aqueous extract of dried calyces (10 mg/kg/b.w)	11 days	↑GSH ↑SOD, ↓CAT, ↓MDA	[130]
*Iris songarica* Schrenk	20 mg/kg/b.w to rats	Ethanolic extract of rhizome (50–300 mg/kg/b.w)	9 days	↑GSH ↑SOD, ↓CAT, ↓MDA	[131]
Melissa Offcinales	20 mg/kg/b.w	Aqueous extract of plant (100–2000 mg/kg/b.w)	14–24 days	↓ALT, ↓AST ↓ALP, ↑GSH	[132]
*Morus nigra*	20 mg/kg/b.w to rats	Ethanolic extract of leaves (500 mg/kg/b.w)	14 days	↓ALT, ↓AST ↓ALP, ↓LDH,	[133]
*Piper betle*	20 mg/kg/b.w to rats	Ethanolic extract of leaves (50–100 mg/kg/b.w)	9 days	↓ALT, ↓AST ↓ALP, ↑GSH	[134]
*Punica granatum*	20 mg/kg/b.w to mice	Ethanolic extract of fruit (0.2 v/v + drinking water)	14 days	↓Bcl‐2 ↓Rho/Cdc42 ↓p‐SAPK/JNK ↑Nrf2	[135]

## 5. Pharmacological Agents in MTX‐LI

There has been a growing focus on various pharmaceutical approaches that have been explored to mitigate MTX‐LI. These interventions include synthetic compounds, antioxidants, and other therapeutic agents that aim to reduce oxidative stress, inflammation, and liver cell damage caused by MTX toxicity. The studies reviewed in Table 2 highlight the potential of these pharmacological agents to protect liver function and support recovery, offering valuable insights into complementary treatments for patients undergoing MTX therapy.

**Table 2:** Role of enzymes, metabolites and pharmaceutical agents in mitigating methotrexate (MTX)‐induced liver injury.

Drug	Dose of methotrexate and animal model	Treatment regimen	Mechanism of action (s)	Parameters and markers evaluated	References
Enzymes					
Aldehyde oxidase (Enzyme)	5 µM to HEPG2 cell lines	1–10 mM	Involved in metabolism of various substrates, detoxify harmful metabolites in liver	↑ cell viability	[137]
Metabolites					
α‐Ketoglutarate (Metabolite)	20 mg/kg/b.w to rats	2 g/kg/b.w (5 days)	Enhances liver detoxification, reduces oxidative stress through metabolic pathways	↑GSH, ↑CAT ↓ALT, ↓AST, ↓ALP ↓LPO	[138]
α‐ lipoic Acid (Antioxidant)	20 mg/kg/b.w to rats	60 mmol/kg/b.w (5 days)	Scavenges free radicals, improves cellular antioxidant defenses through Nrf2/HO‐1	↑GSH ↓ALT ↓AST ↓ALP ↓iNOS ↓TNF‐α ↓MPO ↓ α‐SMA	[139, 140]
Ascorbic acid (Vitamin C)	20 mg/kg/b.w to rats	10 mg/kg/b.w (7 days)	Counters oxidative stress markers by scavenging free radicals	↑GSH, ↑SOD ↑CAT, ↓MDA	[141]
Dexpenthenol (Vitamin B5 derivative)	20 mg/kg/b.w to rats	500 mg/kg/b.w (24 days)	Counters oxidative stress markers by scavenging free radicals	↑GSH, ↑SOD ↑CAT, ↓ALT, ↓AST	[142]
Hydrogen sulfide (gasotransmitter signaling molecule)	20 mg/kg/b.w to rats	56 µmol/kg/b.w (10 days)	Reduces oxidative stress and inflammation, acts as a signaling molecule to protect liver	↑ eNOS, ↑GSH ↓ALT, ↓MDA ↓IL‐6, ↓NF‐κB ↓STAT3	[143]
Melatonin (hormone)	20 mg/kg/b.w to rats	25 mg/kg/b.w (14 days)	Strong antioxidant, modulates oxidative stress and inflammation through PI3K/Akt/mTOR signaling	↑SOD, ↑CAT ↑GSH, ↓AST ↓ALT, ↓MDA	[144, 145]
N‐acetylcysteine (Antioxidant)	20 mg/kg/b.w to rats	50–150 mg/kg/b.w (5 days)	Scavenges free radicals, restores glutathione levels, reduces oxidative stress and inflammation	↑GSH, ↑SOD ↑CAT, ↓MDA	[141, 146]
Taurine (amino acid)	20 mg/kg/b.w to rats	50 mg/kg/b.w (5 days)	Reduces oxidative stress and inflammation in liver by scavenging free radicals	↑GSH, ↓MDA ↓MPO	[147]
Tempol nitroxide (radical scavenger)	20 mg/kg/b.w to rats	30 mg/kg/b.w (10 days)	Radical scavenger, reduces oxidative stress, enhances liver defense mechanisms	↑SOD, ↑CAT ↑GPx, ↓MDA, ↓MPO ↓AST, ↓ALT	[148]
Tranilast (tryptophan metabolite analog)	20 mg/kg/b.w to mice	300 mg/kg/b.w (10 days)	Reduces fibrosis, inflammation, and oxidative stress in liver	↑GSH, ↓MDA ↓AST, ↓ALT, ↓NOx	[149]
Pharmaceutical agents					
Alpelisib (antioxidant)	20 mg/kg/b.w to mice	2.5–5 mg/kg/b.w (5 days)	Antioxidant, anti‐inflammatory, reduces oxidative stress	↑HO‐1 ↑GSH ↓MDA, ↓4‐HNE	[150]
Amifostin (antioxidant)	20 mg/kg/b.w to rats	50 mg/kg/b.w^a^ (7 days)	Reduces oxidative stress by scavenging for and eliminating the DNA‐damaging free oxygen radicals and reactive nucleophile	↑GSH ↑SOD ↑CAT ↓MDA	[141]
Empagliflozin (SGLT2 inhibitor)	20 mg/kg/b.w to mice	10–30 mg/kg/day (7 days)	Reduces oxidative stress, regulates glucose metabolism, reduces inflammation	↑GSH, ↑SOD ↓MDA, ↓AST, ↓ALT	[151]
Enoxaparin (anticoagulant)	20 mg/kg/b.w to rats	180 μg/kg/b.w (10 days)	Anticoagulant, anti‐inflammatory, reduces blood coagulation, inhibits inflammation	↑ eNOS, ↓iNOS, ↓NOSTRIN ↑GSH, ↓fibrin, ↓MDA ↓SOD, ↓CAT	[152]
Liraglutide (GLP‐1 agonist)	20 mg/kg/b.w to rats	2.4 mg/kg/b.w (7 days)	Reduces inflammation, modulates antioxidant activating by Nrf‐2 and pCREB signaling	↑Nrf‐2, ↓TNF‐α ↓IL‐6, ↓COX2, ↓NF‐κB	[153]
Molsidomine (vasodilator)	20 mg/kg/b.w to rats	4 mg/kg/b.w (7 days)	Increases nitric oxide production, reduces oxidative stress and inflammation in the liver	↑SOD, ↑GSH ↑GSH‐Px, ↑CAT	[154]
Montelukast (leukotriene receptor agonist)	20 mg/kg/b.w to rats	10 mg/kg/b.w (10 days)	Reduces leukotriene‐mediated inflammation, inhibits oxidative stress pathways	↑GSH, ↓MPO ↓MDA	[155]
Nevibolol (β‐blocker)	20 mg/kg/b.w to rats	10 mg/kg/b.w (7 days)	Reduces oxidative stress and inflammation, modulates hepatoprotective mechanisms 18through AKT1/Hif1α/ eNOS signaling	↓NO, ↓AST ↓ALT, ↓Total Bilirubin	[156]
Nicorandil (K^+^ channel activator)	20 mg/kg/b.w to rats	3 mg/kg/b.w (14 days)	Protects liver by modulating oxidative stress, inflammatory and apoptotic pathways	↑eNOS, ↓ALT ↓AST, ↓MDA, ↓NOx	[157]
Ramelteon (melatonin receptor agonist)	20 mg/kg/b.w to rats	10 mg/kg/b.w (7 days)	Neuroprotective, anti‐inflammatory, modulates oxidative stress responses	↓IL‐1β, ↓AST ↓AST	[158]
Roflumilast (phosphodiesterase‐4 inhibitor)	5 mg/kg/b.w (4 days) to rats	5 mg/kg/b.w (4 days)	Reduces inflammation and oxidative stress by inhibiting phosphodiesterase‐4	↑GSH, ↓ALT ↓AST, ↓MDA ↓IL‐6	[159]
Sitagliptin (DPP‐4 inhibitor)	20 mg/kg/b.w to mice	10–20 mg/kg/b.w (5 days)	Modulates glucose homeostasis, reduces inflammation, improves insulin sensitivity	↑Nrf‐2, ↓ALP ↓LDH, ↓NF‐κB ↓iNOS	[160]

^a^Administered as a single dose in a 7‐day long experiment.

## 6. Synergistic Effects and Combinations of Pharmaceuticals

### 6.1. Combinational Therapy

Combinational therapy has emerged as an effective strategy for alleviating MTX‐induced hepatotoxicity, utilizing the synergistic effects of multiple compounds to target various pathological pathways involved in liver damage. MTX, a widely used chemotherapeutic agent, causes oxidative stress, inflammation, mitochondrial dysfunction, and apoptosis in the liver, overwhelming the natural repair mechanisms of liver and requiring a multifaceted therapeutic approach. By targeting multiple mechanisms simultaneously, combinational therapies aim to enhance treatment efficacy while minimizing side effects. Several combinations have shown significant hepatoprotective benefits, boosting antioxidant defenses, reducing oxidative stress, and inhibiting inflammatory pathways. For instance, combinations like alpha lipoic acid with Vitamin C and Curcumin with Vitamin C exhibit strong antioxidant properties, neutralizing free radicals and preventing lipid peroxidation to protect liver cells from MTX‐induced damage [161, 162]. Similarly, Omega‐3 complemented with Vitamin C and Niclosamide with Vitamin C target inflammation and oxidative stress, with Vitamin C enhancing the anti‐inflammatory effect [163, 164]. Therapeutic combinations of pentoxifylline with alpha lipoic acid have proven effective in mitigating MTX‐LI by targeting different cellular mechanisms, including reducing lipid peroxidation and enhancing GSH levels [165]. Additionally, combinations like Melatonin with L‐Carnitine focus on mitochondrial protection and cytokine modulation, respectively, providing a broader spectrum of therapeutic effects [166].

Furthermore, the combination of captopril with telmisartan has demonstrated potent effects by inhibiting the renin‐angiotensin‐aldosterone system (RAAS), contributing to reduced fibrosis and inflammation [167] Collectively, these combinational therapies provide a comprehensive strategy, targeting the oxidative, inflammatory, and apoptotic pathways to effectively mitigate MTX‐induced hepatotoxicity as summarized in Table 3. The synergistic effects observed in these therapies underscore the potential of using multi‐targeted approaches in liver protection.

**Table 3:** Role of vitamins and metabolites in combinations in mitigating methotrexate (MTX)‐induced liver injury.

Drug	Dose of methotrexate and animal	Treatment regimen	Treatment duration	Mechanism of action(s)	Parameters of liver injury evaluated	References
α‐Lipoic Acid (ALA) + Vitamin C	20 mg/kg/b.w to mice	ALA (60–120 mg/kg/b. w); Vitamin C (100–200 mg/kg/b. w)	10 days	Counters oxidative stress markers and inflammation	↓ MDA, ↓ALT, ↓ALP, ↓LDH	[161]
Curcumin + Vitamin C	20 mg/kg/b.w to mice	Curcumin (10 mg/kg/b.w); Vitamin C (100 mg/kg/b.w)	10 days	Modulates apoptotic and oxidative stress pathways	↓ MDA, ↓ALT, ↓ALP, ↓LDH	[162]
Omega3 + Vitamin C	20 mg/kg/b.w to mice	Omega 3 (100 mg/kg/b.w); Vitamin C (100 mg/kg/b.w)	10 days	Reduces ROS and LPO, apoptotic and inflammatory pathways	↓ MDA, ↓LDH ↑SOD ↑GSH	[163]
Niclosamide + Vitamin C	20 mg/kg/b.w to mice	Niclosamide (70 mg/kg/b.w); Vitamin C (100 mg/kg/b.w)	10 days	Downregulates mitochondrial dysregulation and activates antioxidant pathways	↓ALT, ↓ALP, ↓LDH	[164]
Melatonin + L‐carnitine	20 mg/kg/b.w to rats hepatocytes	Melatonin (1 mM); L‐carnitine (10 mM)	N/A	Modulates antioxidant, anti‐inflammatory and anti‐apoptotic pathways	↓LPO, ↓NO, ↓TNF‐α, ↓Caspase‐3	[166]
Pentoxifylline + α‐lipoic A (ALA)	20 mg/kg/b.w to rats	Pentoxifylline (50 mg/kg/b.w); ALA (100 mg/kg/b.w)	10 days	Activates antioxidant pathways	↓MDA, ↓NO, ↓BUN, ↓CAT, ↓XO, ↓TNF‐α, ↑SOD, ↑GSH‐Px, ↑DBil	[165]
Captopril + Telmisartan	20 mg/kg/b.w to rats	Captopril (100 mg/kg/b.w); Telmisartan (10 mg/kg/b.w)	7 days	Downregulates expression of inflammatory markers (COX‐2, iNOS)	↓ALT, ↓ALP, ↓AST, ↓MDA, ↓NOx, ↑SOD	[167]
L‐carnitine + Infliximab	20 mg/kg/b.w to rats	L‐carnitine (500 mg/kg/b.w); Infliximab (7 mg/kg/b.w)^a^	5 days	Downregulates Notch1/Hes‐1 signaling	↓TNF‐α, ↓IL‐6, ↓IL‐1β	[168]

^a^Infliximab administered as a single dose.

## 7. Miscellaneous

Apart from the conventional therapies involving phytochemicals and pharmacological agents used in mitigating liver injury, there is growing interest in exploring other unconventional therapies. For instance, human placental extract (HPE) is rich in bioactive molecules like growth factors and cytokines, which help reduce inflammation, enhance tissue repair, and promote regeneration of damaged liver cells [169]. Also, MSCs, through their differentiation potential and secretion of paracrine factors, aid in liver regeneration, immune modulation, and fibrosis reduction [170]. Moreover, beta‐glucan, an immunomodulatory agent, activates immune cells like macrophages, enhancing antioxidant enzyme activity and reducing oxidative stress, leading to improved liver function. Additionally, ursodeoxycholic acid (UDCA) works by stabilizing hepatocyte membranes, reducing bile acid toxicity, and promoting liver regeneration while exerting anti‐inflammatory effects [170]. Lastly, manganese SOD (MnSOD) is an antioxidant enzyme that scavenges harmful superoxide radicals, reduces oxidative stress, and boosts the antioxidant defenses, mitigating hepatocellular damage [171]. Together, these therapies target multiple pathways involved in oxidative stress, inflammation, and liver regeneration, offering significant protective effects against MTX‐LI as summarized in Table 4. Their combined mechanisms of action, including immune modulation, oxidative stress reduction, and cellular regeneration, highlight their therapeutic potential for liver protection in clinical settings.

**Table 4:** Role of miscellaneous agents in countering methotrexate (MTX)‐induced liver injury.

Therapy	Dose of methotrexate	Treatment regimen	Mechanism of action(s)	References
Human placental extract (HPE) (regenerative medicine)	5 mg/kg/b.w (5 days) to rats	10.08 mg/kg/b.w (14 days)	Growth factors, cytokines, and bioactive molecules reduce inflammation, enhance tissue repair, and regenerate damaged liver cells	[169]
Mesenchymal stem cells (MSCs) (cell‐based Therapy)	14 mg/kg/week b. w (14 days) o rats	2 × 10^6^ cells	MSCs differentiate into hepatocyte‐like cells and release paracrine factors that promote tissue repair, modulate immune response, and reduce fibrosis	[172]
Beta‐Glucans (Polysaccharide)	20 mg/kg/ b. w. to rats	50 mg.kg/b.w (5 days)	Activates macrophages and enhances antioxidant enzyme 4 activity, modulating immune responses and reducing oxidative stress	[173]
Ursodeoxyc‐holic acid (UDCA) (bile acid derivative)	20 mg/kg/b.w. to rats	50 mg.kg/b.w (5 days)	Reduces bile acid toxicity, enhances hepatocyte membrane stability, and promotes liver regeneration and anti‐inflammatory effects	[170]
Manganese superoxide dismutase (MnSOD) (antioxidant enzyme)	40 μg/well on human hepatocyte cell line L‐02	Mn‐SOD plasmid trannsfection	Scavenges superoxide radicals, reduces oxidative stress, and enhances liver antioxidant defense systems	[171]

## 8. Challenges and Future Perspectives

The treatment of MTX‐LI, commonly used in chemotherapy and autoimmune disorder, has garnered increasing attention due to the hepatotoxic nature of MTX. Phytochemicals with their array of bioactive properties have shown considerable promise in mitigating liver damage caused by MTX. However, several challenges hinder the effective application and widespread clinical use of phytochemicals and pharmaceutical interventions for this purpose. Addressing these challenges, as well as the potential for future research, can pave the way for improved therapeutic strategies.

One of the primary challenges in exploring phytochemicals for MTX‐LI is the variability in composition of the plant extracts and quality of herbal preparations. Phytochemicals are naturally occurring compounds extracted from plants, but their concentration may differ depending on several factors, including plant species, environmental conditions, harvesting time, and processing methods. This inconsistency can affect the reliability and reproducibility of experimental results. Furthermore, many herbal formulations are used as complex mixtures, which complicates the identification of the specific active compounds responsible for the observed hepatoprotective effects. The standardization of phytochemical extracts remains a significant hurdle to ensuring quality and consistency in clinical applications. Another significant issue is the bioavailability and pharmacokinetics of phytochemicals. While certain phytochemicals exhibit potent hepatoprotective effects in vitro, their bioavailability in vivo can be low due to poor absorption, rapid metabolism, and elimination from the body. Most phytochemicals undergo extensive metabolism in the liver, often resulting in a reduced concentration of the active compound reaching the target organ. Therefore, enhancing the bioavailability and optimizing the pharmacokinetics of these compounds through formulations like nanoparticles or conjugation to other molecules is a crucial area of focus. In preclinical models, species‐specific differences in metabolism can complicate the extrapolation of results to humans. For example, rodents, commonly used in MTX‐LI studies exhibit differences in liver enzyme activity compared to humans. Specifically, CYP2D enzymes, involved in the metabolism of many drugs and are responsible for majority of oxygenation reactions, are present in different isoforms (CYP2D9, CYP2D10, CYP2D11, CYP2D12, CYP2D13, and CYP2D22) in mice but only in a singular form (CYP2D6) in humans. This difference in the distribution of cytochrome P450 families in mice and humans may lead to different pharmacokinetic profiles and relative metabolism rates in animal models, which can skew the translation of data to human clinical settings [6]. However, since MTX is not primarily metabolized by cytochrome P450 enzymes, this specific example is not directly relevant to its hepatotoxicity. A more pertinent challenge is the species‐specific variation in the activity of other metabolic and transport pathways, such as those involving folate enzymes and hepatic transporters, which are more directly implicated in MTX disposition and toxicity. Future studies should focus on characterizing these relevant pathways across species to improve translational predictability. Despite promising results in animal models and in vitro studies, there is a lack of large‐scale, well‐designed clinical trials evaluating the safety and efficacy of phytochemicals and pharmacological agents for attenuating MTX‐LI. Most of the existing studies are limited by small sample sizes, short durations, and variations in study design. To validate the therapeutic potential of phytochemicals, larger, multicentric trials with rigorous methodologies and long‐term follow‐up are required.

Several plant species have not yet been fully explored for their potential benefits in liver diseases, including MTX‐LI. The identification and characterization of novel phytochemicals through advanced screening techniques and high‐throughput assays could lead to the discovery of more effective treatments for MTX‐induced liver toxicity. Another exciting avenue for future research is the application of nanotechnology to improve the delivery and bioavailability of phytochemicals. Nanoparticles, liposomes, and other nanocarriers have been increasingly employed to enhance the solubility and stability of phytochemicals, allowing for more effective delivery to the liver. Such advanced drug delivery systems can also target specific liver cells, minimizing side effects and improving therapeutic outcomes. Incorporating nanotechnology into the development of phytochemical‐based therapies holds great potential in overcoming the limitations of bioavailability and pharmacokinetics. Additionally, personalized medicine approaches are likely to become an integral part of MTX‐LI management since the genetic and phenotypic variability among individuals affects how they metabolize drugs, including MTX and its adjunct therapies. Understanding the genetic makeup and metabolic pathways of patients can help tailor specific treatments to maximize efficacy while minimizing adverse effects. Incorporating phytochemicals into clinical settings for MTX‐LI management requires overcoming several practical challenges. While phytochemicals have demonstrated hepatoprotective properties in laboratory settings, their clinical application must address issues such as standardization, formulation, and dosing. Clinical trials and regulatory approval processes are necessary to ensure that phytochemicals are safe and effective for human use. Unlike conventional pharmaceuticals, many phytochemicals are considered dietary supplements, and their regulatory oversight is often less stringent. Rigorous regulatory frameworks are necessary to ensure that phytochemical‐based therapies are safe, effective, and well‐defined for clinical use in the treatment of MTX‐LI.

## 9. Conclusion

Phytochemicals, pharmacological agents, metabolites, and enzymes have shown significant potential in ameliorating MTX‐LI through their multifaceted mechanisms of action, including the modulation of oxidative stress, inflammation, and fibrosis. These compounds offer promising therapeutic benefits with their ability to target key pathways such as the Nrf2/HO‐1 signaling pathway, reduce lipid peroxidation, and inhibit apoptotic cascades demonstrating their potential as viable candidates for adjunctive therapies in managing MTX‐LI. The diverse range of investigated compounds, from pharmaceutical drugs to natural phytochemicals and endogenous metabolites, highlights a broad potential in combating liver damage caused by MTX. The incorporation of these protective compounds alongside MTX treatment holds considerable promise in clinical settings since their hepatoprotective properties suggest they could be used to mitigate the liver damage caused by MTX that may offer patients dual benefit of treating the underlying condition while preventing or reducing hepatic injuries. However, the clinical application of these approaches requires further validation through large‐scale controlled trials to ensure their safety, efficacy, and consistency across different patient populations. While the results from preclinical studies are promising, more rigorous research are needed to fully understand the potential of phytochemicals in treating MTX‐LI. This includes advancing our knowledge through larger‐scale clinical trials, regulatory toxicology studies, establishing safety and efficacy in humans and overcoming challenges related to formulation, bioavailability, and species‐specific variations in metabolism. Innovations in drug delivery systems such as the use of nanotechnology to enhance the bioavailability of poorly soluble agents, whether phytochemicals or repurposed drugs, will also be important to their future clinical success. Ultimately, continued preclinical, pharmaceutical, and clinical research and drug development strategies will be essential for integrating these hepatoprotective compounds from diverse classes as effective adjuncts to MTX therapy. The integrated approach will provide safer and more effective treatment options for patients suffering from MTX‐induced hepatotoxicity.

## Funding

This study was funded by the United Arab Emirates University (Grants 12M306 and 12R121 to Shreesh Ojha and 12S134, 21S117, and 12S223 to Sameer Mirza).

## Conflicts of Interest

The authors declare no conflicts of interest.

## Data Availability

It is a review article; therefore, data sharing is not applicable to this article, as no datasets were generated during the current study.
